# Esketamine opioid-free intravenous anesthesia versus opioid intravenous anesthesia in spontaneous ventilation video-assisted thoracic surgery: a randomized controlled trial

**DOI:** 10.3389/fonc.2023.1145953

**Published:** 2023-05-31

**Authors:** Qisen Fan, Jinhui Luo, Qianling Zhou, Yaoliang Zhang, Xin Zhang, Jiayang Li, Long Jiang, Lan Lan

**Affiliations:** ^1^ Department of Anesthesia, The First Affiliated Hospital of Guangzhou Medical University, Guangzhou, China; ^2^ Department of Medical Imaging, Guangdong Second Provincial General Hospital, Guangzhou, China; ^3^ National Clinical Research Center for Respiratory Disease and Departments of Thoracic Surgery, The First Affiliated Hospital of Guangzhou Medical University, Guangzhou, China

**Keywords:** opioid-free anesthesia, opioid anesthesia, spontaneous ventilation, video-assisted thoracic surgery, mechanical ventilation

## Abstract

**Background:**

Opioid-free anesthesia (OFA) provides adequate analgesia and can reduce postoperative opioid consumption, but its efficacy in spontaneous ventilation video-assisted thoracic surgery (SV-VATS) has not been demonstrated. We aimed to investigate the hypothesis that OFA could provide the same perioperative pain control as opioid anesthesia (OA), maintain safe and stable respiration and hemodynamics during surgery, and improve postoperative recovery.

**Methods:**

Sixty eligible patients (OFA group: n=30; OA group: n=30) treated between September 15, 2022, and December 15, 2022, at The First Hospital of Guangzhou Medical University were included. They were randomized to receive standard balanced OFA with esketamine or OA with remifentanil combined with sufentanil. The primary outcome was the pain numeric rating score (NRS) at postoperative 24 h, and the secondary outcomes were intraoperative respiratory and hemodynamic data, opioid consumption, vasoactive drug dosage, and recovery in the post-anesthesia care unit and ward.

**Results:**

There was no significant difference in the postoperative pain scores and recovery quality between the two groups. The OFA group had a significantly lower dose of phenylephrine (*P*=0.001) and a lower incidence of hypotension (*P*=0.004) during surgery. The OFA group resumed spontaneous respiration faster (*P*<0.001) and had a higher quality of lung collapse (*P*=0.02). However, the total doses of propofol and dexmetomidine were higher (*P*=0.03 and *P*=0.02), and the time to consciousness was longer (*P*=0.039) in the OFA group.

**Conclusions:**

OFA provides the same level of postoperative pain control as OA, but it is more advantageous in maintaining circulatory and respiratory stability and improving the quality of pulmonary collapse in SV-VATS.

## Introduction

Video-assisted thoracoscopic surgery (VATS) has become a minimally invasive choice for thoracotomy (1, 2). Traditional thoracoscopic surgery usually requires double-lumen endotracheal intubation and mechanical ventilation (MV), which is usually performed with the assistance of opioids and muscle relaxants (3). It is now feasible to perform spontaneous ventilation-VATS (SV-VATS) without intubation in the management of wedge resection (4, 5). SV-VATS is often combined with an intercostal nerve block. Intercostal nerve blocks can reduce the amount of opioids required and preserve spontaneous breathing to avoid respiratory failure due to respiratory-related muscle weakness after surgery (6). Current studies have shown that this technique can accelerate postoperative recovery, shorten hospital stay, and improve patient prognosis (7–9).

Previously, opioids were mostly required for analgesia in SV-VATS, which inevitably resulted in respiratory depression and deep breathing, increasing challenges in intraoperative respiratory management and surgical manipulation (10, 11). Esketamine is a non-opioid drug with a strong analgesic effect and the least possible effect on respiration and circulation. Therefore, it has high potential as an alternative intraoperative analgesic to opioids (12, 13). Studies have shown that opioids promote the production of inflammatory factors and tumor micrometastasis (7, 14, 15). Therefore, opioid-free anesthesia (OFA) has been attempted to explore its effectiveness for SV-VATS.

Several studies have shown that OFA can effectively control pain and reduce perioperative opioid consumption (11, 16). However, interpreting these studies is difficult because the definition of OFA varies between studies and institutions (17). Moreover, there is a lack of high-level clinical evidence on whether OFA is harmful or beneficial in SV-VATS procedures.

Therefore, we aimed to investigate the hypothesis that OFA could provide the same perioperative pain control as opioid anesthesia (OA), maintain the safety and stability of respiration and hemodynamics during surgery, and improve postoperative recovery.

## Materials and methods

### Study design

This study was a prospective, single-center, randomized controlled trial. All patients who underwent wedge resection for non-small-cell lung cancer (NSCLC) in the First Affiliated Hospital of Guangzhou Medical University from September 15, 2022, to December 15, 2022, and satisfied the inclusion and exclusion criteria were included. The study was conducted in accordance with the Helsinki Declaration (revised in 2013). The research scheme and methods were reviewed by the Ethics Committee (2020–69). Before inclusion in the study, all participating patients provided written informed consent.

### Patients

Participants were screened, approached, and recruited by study staff, who evaluated patient eligibility.

The inclusion criteria were age between 18 and 64 years, thoracoscopic resection for NSCLC, single or multiple wedge resections, an American Society of Anesthesiologists score of I–II, a body mass index of 16–25 kg/m^2^, and generally normal cardiopulmonary and other vital organ function indicating the patient could tolerate surgery.

The exclusion criteria were a history of previous thoracic surgery, uncontrolled hypertension, severe coronary artery disease, hepatic and renal insufficiency, hyperthyroidism, visual impairment, allergy to any of the drugs in this study, psychiatric disorders, severe cardiopulmonary impairment, history of chronic pain, or regular opioid use.

Elimination criteria were intraoperative blood loss >15% of the expected circulatory volume, thoracoscopy transferring to thoracotomy, intubation, failure to cooperate, or loss to follow-up.

### Randomization and interventions

Patients were randomized in a 1:1 ratio to either the opioid-free group or the opioid group. Randomization was centralized using the SPSS25 random number generator, and each patient was given a unique randomization number (patient code), using sealed opaque envelopes to reveal the treatment arm on the morning of surgery. Each enrolled patient was grouped in the operating room on the day of surgery, and a designated anesthesiologist performed anesthesia management and intraoperative data collection. The surgeons were blinded to the group allocation of the patients. Postoperative follow-up was performed by study personnel who were trained but not involved in patient care.

## Interventions

### Opioid-free anesthesia group

Anesthesia was induced with dexmedetomidine (0.5 μg/kg for 15 minutes), target-controlled infusion (TCI) of propofol (2–3.5 μg/ml), and intravenous infusion of midazolam (0.05 mg/kg), esketamine (0.5 mg/kg), cisatracurium (0.05 mg/kg), and atropine (0.005 mg/kg). During maintenance of anesthesia, TCI dosages of propofol, esketamine, and dexmedetomidine were 1.5–4 μg/ml, 0.2–0.5 mg/kg/h, and 0.5–1.0 μg/kg/h, respectively. Dexmedetomidine was stopped directly after thoracic closure, and propofol and esketamine were stopped at the end of the procedure.

### Opioid anesthesia group

Anesthesia was induced with dexmedetomidine (0.5 μg/kg for 15 minutes), TCI of propofol (2–3.5 μg/ml), and intravenous infusion of midazolam (0.05 mg/kg), sufentanil (0.2 μg/kg), cisatracurium (0.05 mg/kg), and atropine (0.005 mg/kg). During maintenance of anesthesia, TCI of propofol, remifentanil, and dexmedetomidine were administered at 1.5–4 μg/ml, 0.03–0.08 μg/kg/h, and 0.5–1.0 μg/kg/h, respectively. Dexmedetomidine was stopped directly after the pleural cavity closure, and propofol and remifentanil were stopped at the end of the operation.

Patients in both groups did not inhale volatile anesthetics and received betamethasone 5 mg plus ropivacaine 75 mg diluted to 20 ml for intercostal nerve block, of which 2 ml was injected into each intercostal area above and below the adjacent incision. Five ml of 1% lidocaine was used for vagal nerve block on the surgical side to reduce choking and maintain spontaneous breathing during pulmonary traction.

A third-generation double-tube laryngeal mask airway (LMA) was used for ventilation management. In both groups, if spontaneous breathing was not fully restored after anesthesia, manual ventilation or synchronized intermittent mandatory ventilation (SIMV) was used when the oxygen saturation was less than 94% or the PaCO_2_ was more than 80 mmHg. The tidal volume in SIMV mode was set to 3–5 ml/kg, the peak airway pressure did not exceed 9 mmHg, and the frequency was 12–15 times/min to avoid the dilation of collapsed lungs due to excessive tidal volume. A bispectral index sensor (BIS) was maintained between 45 and 60 during the operation. Dopamine or phenylephrine was administered and recorded when the intraoperative blood pressure decreased to less than 20% of the baseline average. After the operation, the patient was transferred to the post-anesthesia care unit (PACU), and the patient could be sent back to the ward after meeting the departure requirements of the PACU (**Supplementary Table 1**).

Postoperatively, patients in both groups received oral imrecoxib 100 mg twice daily to maintain a resting pain numeric rating score (NRS) ≤ 3. If the resting pain NRS was > 3, tramadol 1 mg/kg was given intravenously as remedial analgesia.

### Surgical process

Patients in both groups were operated on in the same manner. Patients were placed in the lateral position with the upper arm extended and secured on a hand rest. The operative procedure was performed with a single-port VATS, depending on the situation. The thoracoscope was inserted between the 5th and 6th ribs in the posterior axillary line with a soft incision protector over the thoracoscope to protect the skin, subcutaneous tissue, ribs, and pleura. Types of surgery included single or multiple wedge resections. The pulmonary expansion pressure on the operative side was 20 cmH_2_O to detect air leakage in the lung tissue, and a 9F thoracic drainage tube was placed. The thoracic drainage tube was removed postoperatively after reexamination *via* chest radiograph if good lung expansion was observed and there was no obvious air leakage or active bleeding.

### Primary outcome

The primary outcome was pain NRS evaluated at rest within 24 h after surgery.

### Secondary outcomes

The secondary outcomes were intraoperative respiratory and circulatory parameters, opioids consumption, vasoactive drug dose, cardiovascular events, time to resume from spontaneous breathing, degree of lung collapse, incidence of body movements, arterial blood gas analysis, and the patient’s overall recovery in the PACU and ward.

The Campos score criteria for lung collapse includes a classification for complete collapse (I), mostly collapsed (II), and partial or no collapse (III). Spontaneous respiratory recovery time was from the beginning of anesthesia induction to the resumption of spontaneous respiration during surgery. The criteria for nocturnal sleep time include a classification for more than 5 h of continuous sleep (I), continuous sleep time of 2–5 h (II), and less than 2 h of continuous sleep (III).

### Sample size calculation and statistical analysis

The sample size was calculated based on the primary outcome. According to the pretest, the pain NRS at postoperative 24 h was 2.0 ± 1.3 in the OFA group and 3.0 ± 1.3 in the OA group. A sample size of 26 was deemed necessary to achieve a power of 80% with a type I error of 0.05. An additional 15% of participants were included to account for possible loss to follow-up. Therefore, the final sample size was 60 participants (30 in each group).

All primary and secondary data were analyzed according to the intention-to-treat principle. Normality was checked using the Shapiro–Wilk test for continuous variables. We used an independent-sample t-test for continuous variables that were normally distributed and ANOVA for repeated measures or the Friedman test for non-normally distributed data. The χ^2^ test or Fisher’s exact test was used for categorical variables, if appropriate. Recovery-related indexes were drawn using the Kaplan–Meier curve and tested by the log-rank test. We used SPSS version 25.0 (IBM SPSS Inc., Armonk, NY) for all statistical analyses. GraphPad Prism 9.0 software (GraphPad Software Inc., La Jolla, CA) was used for drawing figures. PASS 15.0 (NCSS LLC, Kaysville, UT) was used to calculate the sample size.

## Results

### Patient characteristics

**Figure 1:**
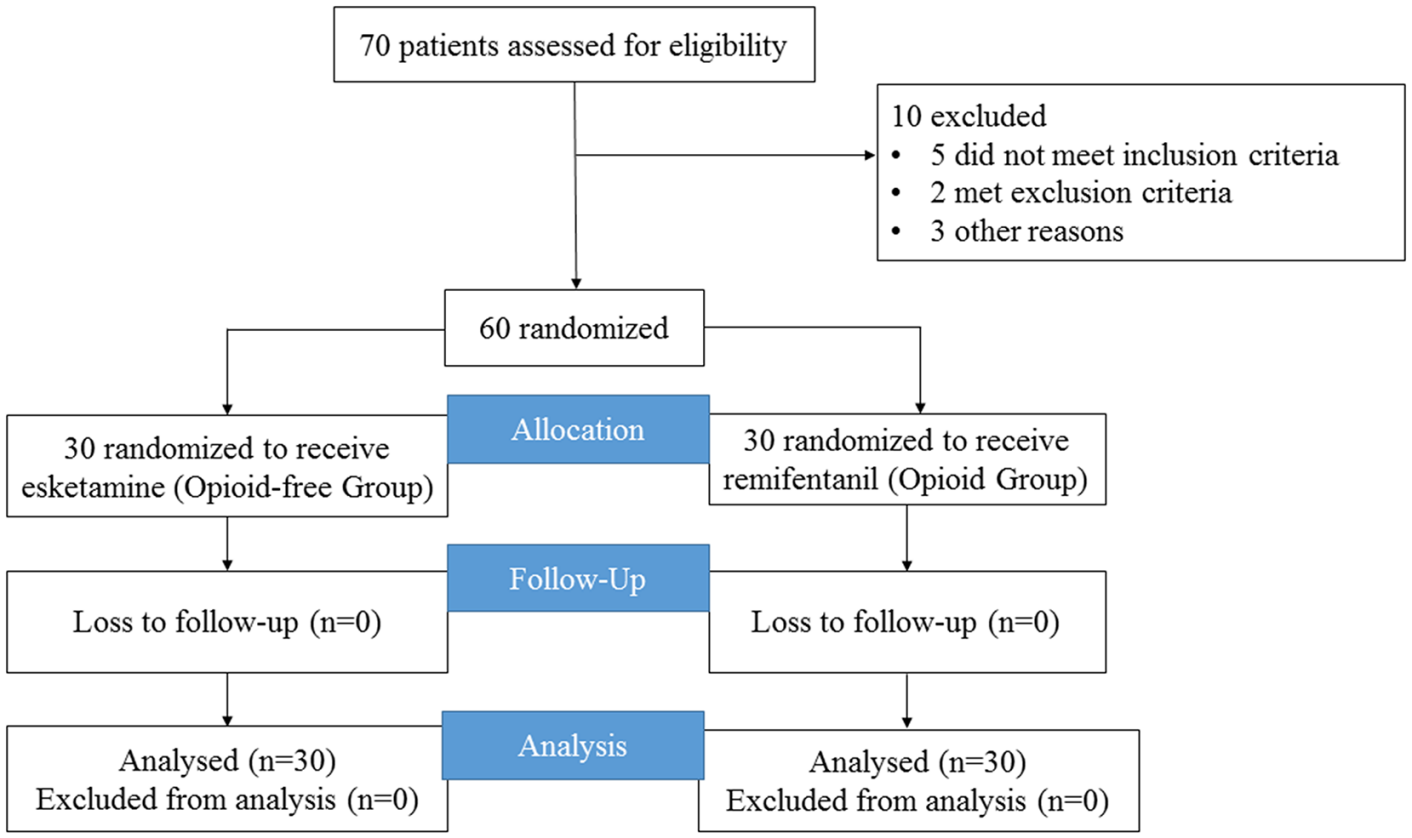
Flowchart of participants.

Sixty of the screened patients were included in the analysis (**Figure 1**). No patients were converted from SV-VATS to MV-VATS. The demographic and clinical characteristics of the two groups were similar (**Table 1**). The intraoperative doses of sufentanil and remifentanil in the control group were 7.5 ± 2.4 μg and 259.7 ± 91.7 μg, respectively. Patients in the OFA group did not receive sufentanil or any other opioids during induction or surgery. Patients in the OFA group had an esketamine dose of 57.9 ± 11.9 mg, with higher doses of propofol (489.7 ± 113.1 mg *vs.* 417.7 ± 137.1 mg, *P*=0.03) and dexmedetomidine (54.4 ± 50.7 μg *vs.* 43.0 ± 15.7 μg, *P*=0.02).

**Table 1:** Characteristics of the patients at baseline.

Characteristic	OA group (n=30)	OFA group (n=30)	*P*-value
Age (years)	48.4 ± 9.3	44.6 ± 6.7	0.07
Gender, n (%)			0.79
Male	11 (36.7)	12 (40.0)	
Female	19 (63.3)	18 (60.0)	
Weight (kg)	58.0 ± 6.9	58.0 ± 8.4	0.96
Body mass index, kg/m^2^	22.6 ± 1.9	21.6 ± 2.4	0.08
ASA physical status*			0.75
I	6	7	
II	24	23	
center ventricular ejection fraction (%)	70.3 ± 5.3	69.4 ± 5.5	0.52
FEV1/FEV **^□^**	94.9 ± 10.5	99.7 ± 9.1	0.06
T stage, n (%)			0.72
T0	4 (13.3)	3 (10.0)	
Tis	2 (6.7)	3 (10.0)	
T1mi	19 (63.3)	22 (73.3)	
T1	5 (16.7)	2 (6.7)	
N stage			1
0	30 (100)	30 (100)	
1	0	0	
M stage			1
0	30 (100)	30 (100)	
1	0	0	
Lymphadenectomy	3 (10.0)	4 (13.3)	1

Regarding intraoperative cardiovascular events, one case of hypertension occurred in each group, but the incidence of hypotension (*P*=0.004) and the intraoperative dose of phenylephrine (*P*=0.001) were significantly higher in the opioid group than in the opioid-free group. The details of the intraoperative data are summarized in **Table 2**.

**Table 2:** Intraoperative data.

Variable	OA group (n=30)	OFA group (n=30)	*P*-value
**Dose of anesthetic drugs** Propofol, mg Esketamine, mg Remifentanil, µg Sufentanil, μg Dexmedetomidine, μg Dose of cisatracurium, mg	417.7 ± 137.1 0 259.7 ± 91.7 7.5 ± 2.4 43.0 ± 15.7 3.3 ± 1.1	489.7 ± 113.1 57.9 ± 11.9 0 0 54.4 ± 50.7 3.6 ± 1.2	0.03* 0.02* 0.29
**Vasoactive drugs** Atropine, mg Dopamine, mg Phenylephrine, μg	0.4 ± 0.1 2.4 ± 6.2 152.7 ± 183.8	0.3 ± 0.2 0.20 ± 0.6 30.0 ± 61.0	0.07 0.06 0.001**
Hypertension **^△^,** n(%)	1 (3.3)	1 (3.3)	1
Hypotension **^▽^**, n(%)	20 (66.7)	9 (30.0)	0.004**
Bradycardia **^○^**, n(%)	7 (23.3)	1 (3.3)	0.06
Duration of surgery, min	56.3 ± 32.9	63.0 ± 29.9	0.42
Duration of anesthesia, min	91.8 ± 40.4	100.0 ± 38.2	0.42
Infusion volume, ml	546.7 ± 130.6	654.3 ± 281.3	0.06
Blood loss, ml	5.4 ± 2.8	9.1 ± 17.5	0.27

The data are presented as means ± SD. *P<0.05, **P<0.01.

^△^Hypertension was defined as the mean arterial blood pressure higher than 90 mmHg. ^▽^Hypotension was defined.

as mean arterial blood pressure lower than 65 mmHg. ^○^Heart rate less than 50 beats/min.

### Primary outcome

Time significantly affected the pain score (*P*<0.001). Comparison within groups showed that the NRS at 6 h (*P*<0.001) and 24 h (*P*<0.05) was higher than NRS 2 h after surgery. The NRS was higher at 6 h than at 24 h after surgery (*P*<0.01), but the NRS was not significant between the groups (**Table 3**).

**Table 3:** Resting numerical rating scale.

Variable	2h	6h	24h	Total
**OA group** **(n=30)**	0.9 ± 0.8	1.7 ± 0.9	1.2 ± 0.7	1.3± 0.13
**OFA group** **(n=30)**	1.0 ± 1.4	1.8 ± 1.2	1.4 ± 1.1	1.4 ± 0.13
**Total**	0.95 ± 0.14	1.73 ± 0.14***	1.30 ± 0.12*^θθ^	

The data are presented as mean ± standard deviation. Comparison with 2 hours after operation.

*P<0.05, ***P<0.001. Comparison with 6 hours after operation, ^θθ^P<0.01.

### Secondary outcomes

**Figure 2:**
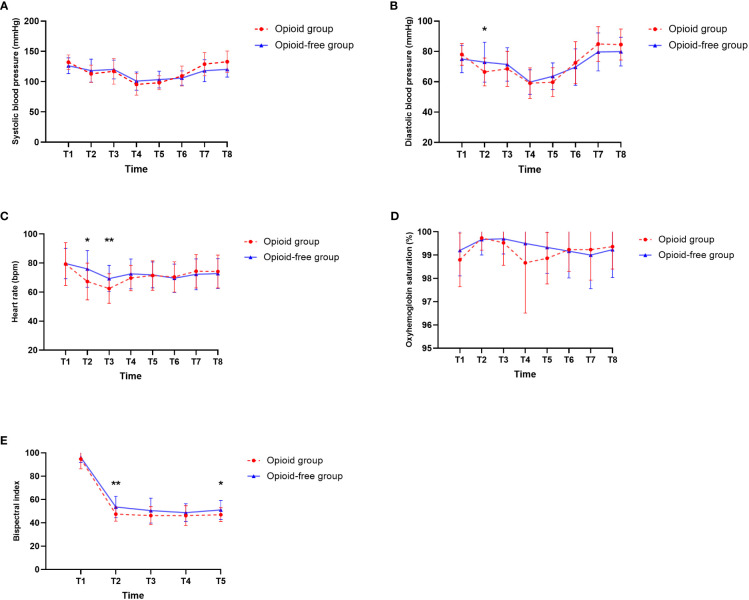
Intraoperative hemodynamic data, anesthetic depth. **(A)** Systolic blood pressure. **(B)** Diastolic blood pressure. **(C)** Heart rate. **(D)** Oxyhemoglobin saturation. **(E)** Bispectral index. T1, Baseline. T2, After the insertion of the laryngeal mask. T3, At the time of incision. T4, During the wedge resection. T5, At the end of surgery. T6, After transfer to PACU. T7, Immediately after removal of the laryngeal mask. T8, When ready to leave the PACU. *P<0.05, **P<0.01.

In the comparison of intraoperative hemodynamics between the two groups, heart rate, diastolic blood pressure, and BIS values at T2, heart rate at T3, and BIS values at T5 were significantly higher in the OFA group than in the OA group (**Figure 2**, *P<*0.05). There were no significant differences in heart rate, diastolic blood pressure, and BIS values at any other observation periods. There were no significant differences in systolic blood pressure and oxyhemoglobin saturation at T1 to T8. The spontaneous respiratory rate was significantly faster in the OFA group than in the OA group both in wedge resection and after closing the pleura (*P<*0.001), but with no significant difference in tidal volume between the two groups (**Table 4**).

**Table 4:** Intraoperative ventilation characteristics.

Variable	OA group (n=30)	OFA group (n=30)	*P*-value
In wedge resection			
Spontaneous breathing rate (times/min)	7.5 ± 5.8	15.0 ± 4.5	<0.001***
Tidal volume (ml)	124.7 ± 94.9	147.7 ± 61.6	0.27
After closing pleura			
Spontaneous breathing rate (times/min)	12.6 ± 2.9	17.5 ± 3.6	<0.001***
Tidal volume (ml)	240.8 ± 99.5	233.6 ± 57.0	0.73

The data are presented as mean ± standard deviation or n (%). ***P<0.001.

There were no significant differences in the indexes of arterial blood gas analysis at 5 min after anesthesia, immediately after wedge resection, and 5 min after removal of the laryngeal mask, but the oxygenation index was higher in the OFA group than in the OA group immediately after wedge resection (*P*=0.018). The arterial partial pressure of carbon dioxide was higher than 80 mmHg in three patients (10.0%) in the OA group and seven patients (23.3%) in the OFA group, but the oxygen saturation of the patients was maintained above 94%, and these individuals received SIMV-assisted ventilation (**Table 5**).

**Table 5:** Intraoperative arterial blood gas indicators.

Characteristic	OA group (n=30)	OFA group (n=30)	*P*-value
**Oxygenation index (mm Hg)** After anesthesia Immediately after wedge resection After awake 5min	453.8 ± 84.0 261.2 ± 119.5 386.1 ± 197.2	458.2 ± 102.6 326.8 ± 86.2 412.9 ± 160.8	0.86 0.018* 0.57
**Oxygen saturation (%)** After anesthesia Immediately after wedge resection After awake 5min	98.8 ± 1.1 98.7 ± 2.2 99.2 ± 0.9	99.2 ± 1.1 99.5 ± 0.9 99.0 ± 1.4	0.17 0.05 0.51
**Arterial carbon dioxide tension (mm Hg)** After anesthesia Immediately after wedge resection After awake 5min	44.4 ± 4.5 75.8 ± 46.8 47.2 ± 4.5	45.3 ± 7.6 71.7 ± 10.1 47.0 ± 4.4	0.59 0.65 0.87
**Arterial carbon dioxide tension >80 mmHg, n(%)** Yes No	3 (10.0) 27 (90.0)	7 (23.3) 23 (76.7)	0.17
**pH** After anesthesia Immediately after wedge resection After awake 5min	7.4 ± 0.03 7.2 ± 0.06 7.3 ± 0.03	7.4 ± 0.05 7.2 ± 0.07 7.3 ± 0.03	0.64 0.67 0.87

Values are presented as mean ± standard deviation or n (%). Percentages are calculated for the whole population. *P<0.05.

The levels of intraoperative lung collapse were higher (*P*=0.02), and the time of resumption of spontaneous breathing was shorter (*P*=0.001) in the OFA group than in the OA group. There was no significant difference in cases of intraoperative somatic motion between the two groups.

In the PACU, the time to consciousness was significantly longer in the OFA group (*P*=0.039). There were no significant differences in the comparison of the occurrence of dizziness, cognitive dysfunction, pneumonia, pulmonary atelectasis, and duration of nocturnal sleep on the first and second postoperative days between the two groups (*P*>0.05) (**Table 6**). Remedial analgesia also showed no difference between the two groups (*P*>0.05) (**Table 7**).

**Table 6:** Secondary outcome analyses.

Variable	OA group (n=30)	OFA group (n=30)	*P*-value
Campos score, n (%)			0.02*
I	17 (56.7)	26 (86.7)	
II	13 (43.3)	4 (13.3)	
III	0	0	
Time to resume from spontaneous breathing^Ψ^, min	38.7 ± 13.7	24.1 ± 9.4	＜0.001***
Body movement, n (%)	3 (10.0)	9 (30.0)	0.053
Time to consciousness in the PACU, min	23.6 ± 13.3	31.8 ± 16.8	0.039*
Length of stay in the PACU, min	74.5 ± 20.1	74.0 ± 22.5	0.92
Postoperative Day 1, n (%)			
Dizzy	0	1 (3.3)	1.00
Cognitive dysfunction	0	0	1.00
Pneumonia / Atelectasis	1 (3.3)	3 (10.0)	0.61
Nocturnal sleep time			0.19
I	2 (6.7)	5 (16.7)	
II	13 (43.3)	17 (56.7)	
III	15 (50.0)	8 (26.7)	
Postoperative Day 2, n (%)			
Dizzy	0	0	1.00
Cognitive dysfunction	0	0	1.00
Pneumonia / Atelectasis	1 (3.3)	0	1.00
Nocturnal sleep time			0.80
I	13 (43.3)	15 (50.0)	
II	17 (56.7)	15 (50.0)	
III	0	0	

^Ψ^Spontaneous respiratory recovery time was from the beginning of anesthesia induction to the resuming spontaneous respiration during operation. The data are presented as mean ± standard deviation or n (%). *P<0.05, ***P<0.001.

**Table 7:** Postoperative salvage analgesia.

Time	OA group (n=30)	OFA group (n=30)	*P*-value
2h	0	2 (6.7)	0.49
6h	3 (10.0)	9 (30.0)	0.05
24h	5 (16.7)	5 (16.7)	1.00

The data are presented as mean ± standard deviation or n (%).

**Figure 3:**
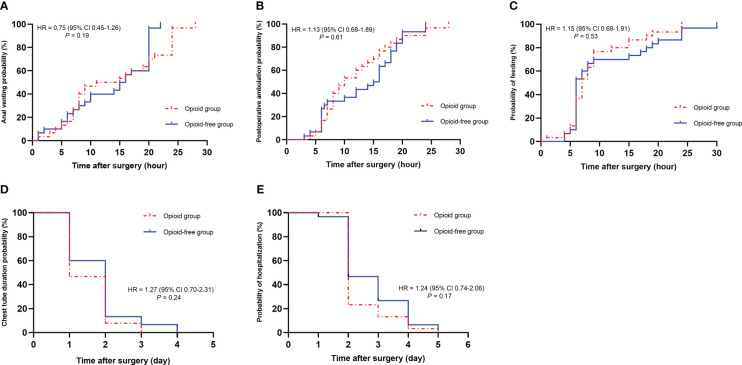
Recovery-related evaluation. **(A)**, Anal venting. **(B)**, Postoperative ambulation. **(C)**, Feeding times. **(D)**, Chest tube duration. **(E)**, Hospitalization.

No significant differences were observed regarding postoperative recovery between the two groups, including anal venting times (14.2 *vs.* 13.5 h, *P*=0.19) (**Figure 3A**), postoperative ambulation (*P*=0.61) (**Figure 3B**), feeding times (*P*=0.53) (**Figure 3C**), chest tube duration (*P*=0.24) (**Figure 3D**), and hospitalization (*P*=0.17) (**Figure 3E**).

## Discussion

In this single-center randomized trial, compared with OA, OFA demonstrated similar pain control in the postoperative period. Moreover, it had more stable hemodynamics. The incidence of hypotension was lower, and the use of phenylephrine was significantly reduced. The quality of pulmonary collapse was better, and the time of resumption of spontaneous respiration was significantly shortened with OFA compared to OA. A higher oxygenation index was observed in the OFA group after wedge resection, possibly due to the mild respiratory excitatory and bronchodilatory effects of esketamine. However, in the OFA group, the intraoperative doses of propofol and dexmetomidine were significantly increased, and the recovery time in the PACU was significantly prolonged. There was no significant difference in postoperative complications and rehabilitation between the two groups.

Traditionally, VATS was performed by double-lumen endotracheal intubation for one-lung ventilation. Although endobronchial intubation allows for lung collapse on the side of the operation and gives the surgeon good maneuvering space, it is associated with complications, such as sore throat and hoarseness (8, 18). MV can also cause or aggravate volutrauma, barotrauma, atelectrauma, and biotrauma (19, 20). The application of SV-VATS has been proven in various thoracic surgeries, such as lobectomy, wedge resection, and tracheal carina reconstruction (4, 5, 7, 9). Spontaneous breathing in thoracic surgery can avoid the complications caused by tracheal intubation and lung injury induced by MV, even lead to residual relaxation (9, 21, 22). Intraoperative regional anesthesia can significantly reduce the dosage of opioids, which is important given that opioids have been proven to demonstrate tumor-promoting activity *in vitro* and accelerate tumor progression through immunosuppression, angiogenesis, and tumor cell migration (23). Previous clinical studies have confirmed that SV-VATS can lead to better survival outcomes than MV in invasive NSCLC (24). Moreover, a number of clinical studies have demonstrated that this method of anesthesia can promote rapid postoperative recovery (8, 22). Whether OFA in spontaneous breathing thoracoscopic surgery can bring more benefits to patients with NSCLC was the key point in that study.

SV-VATS still requires opioid drugs such as remifentanil, but OFA completely avoids the use of opioids during surgery. In this study, the postoperative scores of NRS were less than 3 in the two groups. The main reason may be that the two groups used long-acting hormone mixed with ropivacaine for intercostal nerve block, and some studies have shown that long-acting hormone can significantly prolong the time of analgesia (25–27). No significant differences were observed in NRS scores and rescue analgesia rates between the two groups at the same time after surgery. The pain scores were consistent with a recent meta-analysis by D’Amico that found no difference in pain scores between OFA and OA at 24 h after thoracic surgery (MD -1.69 [-3.82,0.43]; *P* = 0.12) (28). The NRS was the highest at 6 h after surgery due to the weakening of the analgesic effect of the intercostal nerve block and the stimulation of the pleura by the thoracic drainage tube. After the chest tube was removed 24 h after surgery, the degree of pain reduced, and the pain score was lower than that at 6 h after surgery, improving comfort and facilitating the postoperative lung rehabilitation training. Many regional nerve block methods can provide postoperative analgesia in thoracic surgery, such as epidural nerve block, thoracic paravertebral nerve block, and anterior serratus block. However, surgeons have also advocated intercostal nerve block, and the injection is performed by thoracic surgeons under direct thoracoscopic view, making it easy to operate and providing effective treatment. Therefore, regional nerve block was still used the intercostal nerve block method in this study. Esketamine demonstrates quasi-sympathetic activity in pharmacodynamics, and blood pressure decreases significantly after intercostal nerve block in the control group. The possible reason is that the local anesthetic solution blocks the sympathetic nerve along the parietal pleura, leading to decreased blood pressure. It is precisely because of the excitatory effect of esketamine on the circulatory system that the hemodynamics in the opioid-free group was more stable, the incidence of hypotension was reduced, and the amount of phenylephrine used during surgery was also reduced. It was similar to the Forget study that found that ketamine had no significant effect on heart rate but significantly reduced blood pressure variability (29).

At present, most of the commonly used anesthetic drugs can reduce brain metabolism and inhibit brain electrical activity. Esketamine increases the metabolic rate of the brain and inhibits the activity of the cerebral cortex, but the activity of the subcortical structure is enhanced. Some patients have muscle tension, which was verified in this study, and there were higher BIS values in the OFA group than in the OA group. Before completing the intercostal nerve block under direct vision, we increased the infusion of propofol and esketamine to deepen the sedation and enhance analgesia. However, the incidence of intraoperative body movement in the OFA was higher than in the opioid group, possibly due to the sensory-motor separation of esketamine (30). The process of pulmonary collapse during surgery is divided into two main stages. The first stage is the period of rapid lung collapse, due to the opening of the pleural cavity, and the inherent elastic retraction force of the lung tissue promotes lung collapse. The second stage is the period of gas absorption in the lung, in which the residual gas in the lung is diffused and absorbed by itself. Esketamine has the effect of dilating bronchial smooth muscle and improving pulmonary compliance, which promotes gas expulsion in the lung. In addition, the excitatory effect of esketamine in the circulatory system can accelerate the heart rate and increase cardiac output. The increase of blood volume per unit time through the pulmonary circulation also benefits gas absorption in the lung, enhancing the quality of lung collapse in the OFA group. The recovery time in the PACU was significantly prolonged in the OFA group, similar to the results of several studies that found that opioid-sparing anesthesia prolongs the recovery time of patients and increases the dose of sedatives (17, 31). The rate of continuous infusion of esketamine did not exceed 0.6 mg/kg/h during surgery, and the dosage of propofol and dexmedetomidine were significantly higher in the OFA group, which may delay awakening but not increase the total duration of stay in the PACU.

It is worth noting that, although the OFA strategy can avoid the slow down of gastrointestinal peristalsis caused by opioids such as sufentanil and remifentanil, this did not significantly shorten the anal exhaust time of patients. It was speculated that, first, both groups initiated regional nerve block to decrease the dosage of opioids during surgery. Second, the intraoperative analgesia was maintained by a rapidly metabolizing drug, such as remifentanil, in the opioid group, which would not weaken intestinal function after surgery. Finally, there was no significant difference in anal venting time between the two groups, which enabled the implementation of rapid rehabilitation strategies to encourage patients to eat and ambulate early. There was no significant difference in postoperative complications and recovery quality between the two groups.

There are several limitations to this study. First, the degree of analgesia is mainly determined by the change in hemodynamics and the values of BIS in SV-VATS. Thus, using an analgesia monitor would have provided a more objective measure. Second, to make the study more controllable, we mainly selected patients requiring single or multiple wedge resections, and there was no further comparison of other types of surgery. Because this type of thoracic surgery has less procedural heterogeneity, thereby minimizing the influence of differences in surgical procedures between surgeons, it can provide the best perspective for comparing these two different anesthetic modalities. Finally, this was a single-center study, and a larger sample of data is needed to confirm the findings regarding OFA.

## Conclusion

Our findings indicated that compared with OA, OFA provides similar postoperative pain control and recovery quality, but the respiration and circulation were more stable, and the quality of lung collapse was higher. However, this increased the dosage of propofol and dexmedetomidine and prolonged the return to consciousness. Future clinical trials should aim to identify the most effective OFA regimen and pay attention to chronic pain and tumor recurrence after surgery.

## Data availability statement

The raw data supporting the conclusions of this article will be made available by the authors, without undue reservation.

## Ethics statement

The studies involving human participants were reviewed and approved by the Institutional Review Board of the First Affiliated Hospital of Guangzhou Medical University. The patients/participants provided their written informed consent to participate in this study.

## Author contributions

Conception and design: QF, LL, and LJ. Administrative support: LL. Provision of study materials or patients: QF, JHL, YZ, and XZ. Collection and assembly of data: QF, JHL, YZ, and XZ. Data analysis and interpretation: QF, QZ, and JHL. Manuscript writing: All authors. All authors contributed to the article and approved the submitted version.
