# Supplementary material for: Esketamine opioid-free intravenous anesthesia versus opioid intravenous anesthesia in spontaneous ventilation video-assisted thoracic surgery: a randomized controlled trial
Source: Front Oncol. 2023 May 31;13:1145953. doi: 10.3389/fonc.2023.1145953 (PMC10266098; doi:10.3389/fonc.2023.1145953)
Supplement: Supplementary file 1 [file Table_1.docx]

| **Variable** | **score** |
| --- | --- |
| **Sobriety degree** |  |
| Fully awake | 2 |
| Respond to stimuli | 1 |
| No response to stimuli | 0 |
| **Degree of airway patency** |  |
| Coughing as instructed | 2 |
| Independently maintain the patency of the respiratory tract | 1 |
| Need respiratory support | 0 |
| **Limb mobility** |  |
| Do conscious physical activities | 2 |
| Physical unconscious activity | 1 |
| No movement of the limbs | 0 |

**Supplementary Table 1.** Steward score as the criteria for leaving the PACU.

The total score of the three items were six. When the patient's score were greater than four, transfer out of PACU can be considered.
